# Association between tumor genomic mutations and the risk of PD-1 inhibitor-induced hypophysitis: a retrospective cohort study

**DOI:** 10.3389/fendo.2026.1845186

**Published:** 2026-06-08

**Authors:** Yuanyuan Zheng, Yizhen Chen, Wei Lin, Le Min

**Affiliations:** 1 Division of Endocrinology, Diabetes and Hypertension, Brigham and Women’s Hospital, Harvard Medical School, Boston, MA, United States; 2 Department of Geriatric Medicine, Fujian Key Laboratory of Geriatrics Diseases, Fujian Provincial Center for Geriatrics, Fujian Provincial Hospital, Fuzhou University Affiliated Fujian Provincial Hospital, Fuzhou, Fujian, China; 3 Department of Thoracic Surgery, Fujian Provincial Hospital, Fuzhou University Affiliated Fujian Provincial Hospital, Fuzhou, Fujian, China; 4 Department of Endocrinology, Fujian Provincial Hospital, Fuzhou University Affiliated Provincial Hospital, Fuzhou, Fujian, China

**Keywords:** hypophysitis, immune checkpoint inhibitors, PD-1 inhibitors, tumor genomic mutations, tumor mutational burden

## Abstract

**Background:**

Immune checkpoint inhibitors (ICIs) can cause immune-related adverse events (irAEs), with hypophysitis being a serious endocrine toxicity that often requires hormone replacement and poses a risk of adrenal crisis. Biomarkers for predicting ICI-induced hypophysitis are lacking.

**Methods:**

This retrospective, single-center cohort study used the Research Patient Data Registry (RPDR) to identify adults with breast cancer, lung cancer, renal cell carcinoma, or melanoma who received PD-1 inhibitor monotherapy (pembrolizumab or nivolumab) between January 1, 2000, and May 29, 2024. The final cohort included 82 patients with available tumor genomic profiling data, including 26 who developed hypophysitis and 56 matched PD-1 inhibitor-treated controls who did not develop hypophysitis. Tumor mutational burden (TMB) and tumor gene mutation profiles were compared between groups.

**Results:**

Genomic analysis showed that the hypophysitis group had a higher overall tumor mutational burden (6.02 vs. 5.19 mut/Mb, *P* = 0.002). Furthermore, we identified significantly high rate of mutations in the genes *BABAM1* (18.2% vs. 0%, *P* = 0.014), *KDM5C* (18.2% vs. 0%, *P* = 0.014), *CDH4* (19.2% vs. 1.8%, *P* = 0.018), and *TAL1* (19.2% vs. 1.8%, *P* = 0.018) in the hypophysitis group. In contrast, *PAXIP1* mutations were more common in the non-hypophysitis group (19.6% vs. 0%, *P* = 0.037), suggesting a potential protective role.

**Conclusions:**

This exploratory study identifies a tumor genomic profile associated with PD-1 inhibitor-related hypophysitis. Higher TMB and enrichment of selected tumor mutations may reflect a tumor immunogenicity state that predisposes to pituitary autoimmunity under PD-1 blockade. These findings provide a hypothesis-generating genomic framework for future studies of endocrine irAE risk stratification.

## Introduction

Immune checkpoint inhibitors (ICIs) are monoclonal antibodies targeting programmed cell death protein-1 (PD-1), its ligand PD-L1, or cytotoxic T-lymphocyte-associated protein 4 (CTLA-4). These therapeutic agents have revolutionized the treatment landscape for advanced malignancies across a broad range of tumor types (1, 2). However, non-specific immune activation triggered by ICI therapy can induce a heterogeneous spectrum of clinically meaningful toxicities, collectively termed immune-related adverse events (irAEs) (3). Endocrine organs are commonly involved, and ICI-induced hypophysitis is a clinically important toxicity because unrecognized ACTH deficiency can lead to adrenal crisis and long-term hormone replacement (4–6). The frequency and phenotype of hypophysitis vary substantially by ICI class. Hypophysitis is most commonly associated with CTLA-4 blockade and combination CTLA-4/PD-1 therapy, whereas PD-1 inhibitor-associated hypophysitis is less frequent but remains clinically important because it often presents with isolated corticotroph deficiency and may lack typical MRI abnormalities (7–9). These biological and clinical differences support focused evaluation of PD-1 inhibitor-associated hypophysitis as a distinct endocrine irAE phenotype.

Cancer is fundamentally a genomic disease. Tumor genomic characteristics are increasingly utilized to predict the therapeutic efficacy of ICIs (10, 11). Genomic testing and sequencing technologies (GTST) are widely employed in oncology for risk stratification, therapeutic selection, and prediction of treatment response (10, 12). While the role of GTST in guiding targeted therapies is well-established, its utility in predicting immune-related endocrine toxicities, particularly ICI-induced hypophysitis, remains inadequately investigated and poorly defined.

This retrospective study analyzes clinical and tumor genomic data from patients who developed hypophysitis following PD-1 inhibitor therapy, aiming to identify tumor genomic correlates of increased pituitary autoimmunity. The objective is not to prove that individual variants directly alter protein function in the pituitary, but rather to determine whether tumor mutation profiles and TMB are associated with susceptibility to PD-1 inhibitor-induced hypophysitis. These findings may support individualized endocrine monitoring strategies during immunotherapy.

## Materials and methods

### Study objectives

The primary objective was to evaluate the association between tumor gene mutation profiles, tumor mutational burden (TMB), and the occurrence of PD-1 inhibitor-induced hypophysitis. Because CTLA-4 inhibitor-associated hypophysitis and PD-1 inhibitor-associated hypophysitis may differ in incidence, clinical phenotype, and immune mechanism, this study intentionally focused on patients treated with PD-1 inhibitor monotherapy to reduce biological heterogeneity.

### Study design and patient population

Patients with breast cancer (BRCA), lung cancer (LUNCA), melanoma (MELA), or renal cell carcinoma (RCC) who received PD-1 inhibitors (pembrolizumab or nivolumab) and subsequently developed hypophysitis were identified from the Research Patient Data Registry (RPDR) between January 1, 2000, and May2024, 2024 following by medical record review.

**Figure 1:**
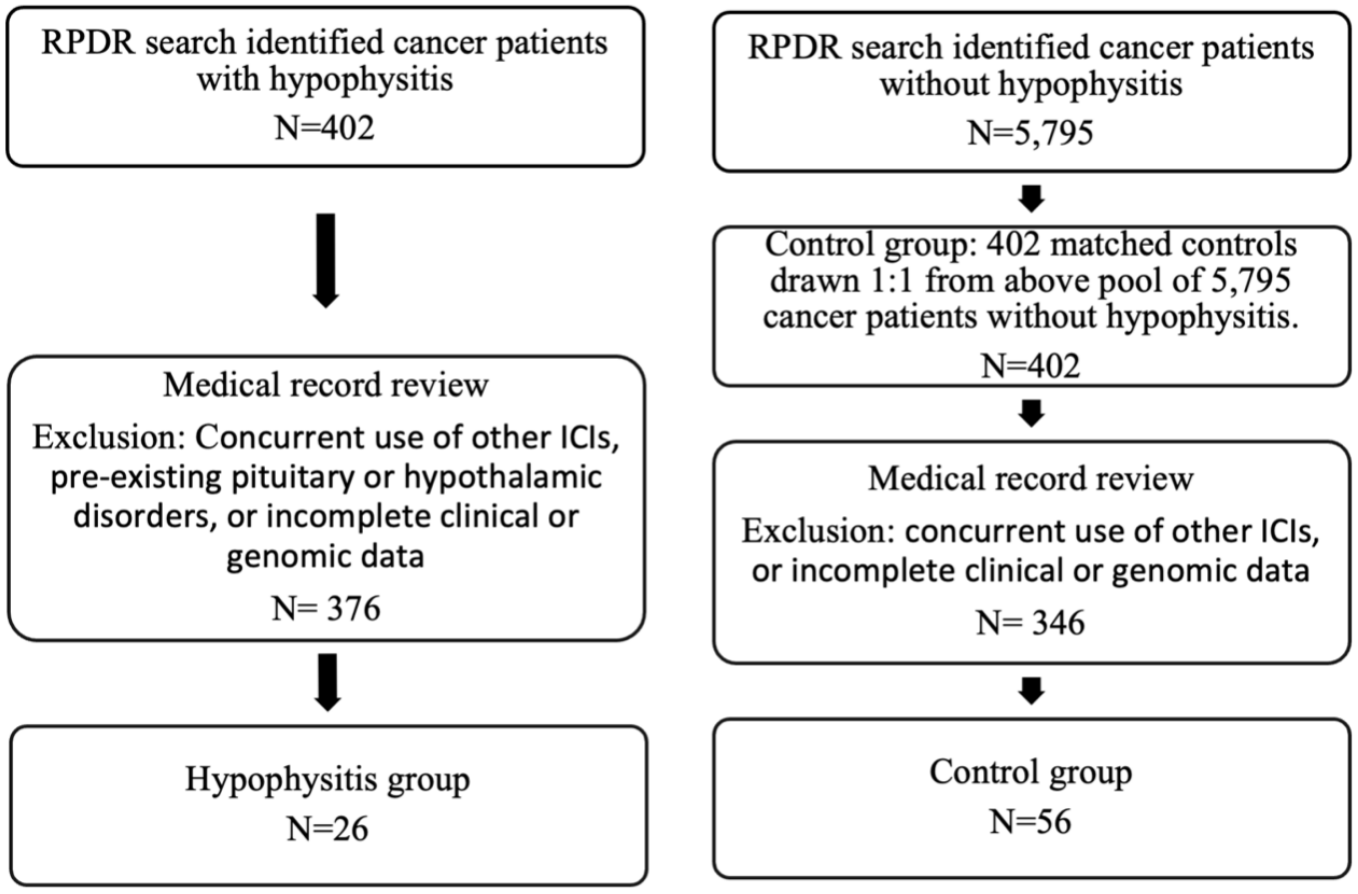
Flowchart of the study.

For comparison, a control cohort of PD-1 inhibitor treated patients without hypophysitis was generated in two stages. First, the RPDR search yielded 5,795 patients with the same cancer types who underwent PD-1 inhibitor therapy without subsequent hypophysitis. Second, 1:1 matching with the hypophysitis group was performed using the RPDR integrated matching tool, based on age, sex, race, and overall health status. Comprehensive medical record reviews confirmed diagnostic accuracy, treatment history, and clinical outcomes. Only patients with available tumor genomic profiling data were included in the final analysis (**Figure 1**).

Eligible patients were adults (≥18 years) with a histologically or cytologically confirmed diagnosis of breast cancer, lung cancer, melanoma, or renal cell carcinoma who had received at least one dose of pembrolizumab or nivolumab. Inclusion criteria required the availability of comprehensive tumor genomic profiling obtained from a validated next-generation sequencing platform, along with complete clinical documentation, including laboratory and imaging data. Patients were required to have a minimum of six months of clinical follow-up after initiation of anti-PD-1 therapy. For those in the hypophysitis group, a confirmed diagnosis of hypophysitis within this follow-up period was mandatory. Patients were excluded if they were younger than 18 years, received concurrent treatment with other immune checkpoint inhibitors, had pre-existing pituitary or hypothalamic disorders (including pituitary adenoma, primary or secondary hypophysitis, or other hypothalamic–pituitary axis diseases), or lacked essential clinical or genomic information such as treatment timelines or standardized follow-up data.

### Cohort stratification

Hypophysitis Group: This cohort consisted of patients who developed immune-related hypophysitis after initiation of PD-1 inhibitor therapy. The diagnosis was confirmed by an endocrinologist according to the Endocrine Society’s consensus guidelines (5). Diagnostic criteria included characteristic clinical symptoms, biochemical evidence of anterior pituitary hormone deficiencies, and/or abnormal pituitary MRI findings. Control Group: This cohort included patients who received PD-1 inhibitor therapy for at least six months (or until discontinuation for any reason) and did not develop hypophysitis of any grade during the entire observation period.

This study was approved by the Mass General Brigham Institutional Review Board and conducted in accordance with the principles of the Declaration of Helsinki. The IRB protocol number is 2023P001615 (MGB/RPDR).

### Genomic analysis

Tumor molecular profiling was performed using OncoPanel, a targeted next-generation sequencing (NGS) panel developed at Brigham and Women’s Hospital and Dana-Farber Cancer Institute (13). OncoPanel is a custom hybrid capture-based assay designed for comprehensive genomic profiling of cancer specimens. The gene panels incorporate capture probes for 275–447 cancer-associated genes, as well as intronic regions of 60 genes for rearrangement detection. This includes the genes listed in **Table 1** (BABAM1, KDM5C, CDH4, TAL1, PAXIP1), in addition to other commonly mutated oncogenes and tumor suppressor genes (14, 15). Because paired normal tissue sequencing was not performed, the identified variants are described as tumor genomic mutations rather than definitively somatic mutations. The available clinical reports provided gene-level mutation status, but not uniform variant-level annotation, variant allele frequency, protein change, or population allele frequency across all patients.

**Table 1:** Profile of genetic mutation.

Gene symbol	Full name	Chromosomal location	Biological function/Pathway
BABAM1	BRISC and BRCA1-A complex member 1	19p13.11	DNA double-strand break repair; NF-kB signaling regulation
KDM5C	Lysine demethylase 5C	Xp11.22	Histone H3K4 demethylation;chromatin remodeling and transcriptional regulation
CDH4	Cadherin-4	20q13.33	Calcium-dependent cell-cell adhesion
TAL1	TAL bHLH transcription factor 1	1p33	Key transcription factor in hematopoietic development
PAXIP1	PAX interacting protein 1	7q36.2	DNA damage repair (53 BP1 co-factor); transcriptional regulation

### Variables and measurements

Baseline clinical data were extracted from the Epic electronic health record (EHR), including age, race, ethnicity, and body mass index (BMI). Race was categorized as White, Black or African American, Asian, Multiracial, or Other, and ethnicity was classified as Hispanic or non-Hispanic. Treatment-related data, including initiation and discontinuation dates of PD-1 inhibitor therapy and dosing history, were collected, along with the timing of hypophysitis onset. Laboratory results at the time of hypophysitis diagnosis were recorded, including morning cortisol, serum adrenocorticotropic hormone (ACTH), electrolytes, thyroid function tests, and sex hormone levels. Brain and pituitary MRI findings, as well as medications administered for hypophysitis management and hormone replacement regimens, were also reviewed.

### Statistical analysis

Categorical variables are presented as frequencies and percentages, and group comparisons were performed using Pearson’s chi-square test, likelihood ratio test, or continuity correction test as appropriate. Continuous variables are summarized as medians and interquartile ranges (IQR). All analyses were conducted using SPSS statistical software (version 25, SPSS Inc., Chicago, IL, USA), and a P-value of <0.05 was considered statistically significant.

## Results

### Clinical characteristics

After application of exclusion criteria, the final study population comprised 26 patients in the hypophysitis group and 56 in the matched non-hypophysitis control group (**Figure 1**).

There were no statistically significant differences between the two groups with respect to sex, race, ethnicity, cancer type, or PD-1 inhibitor treatment, indicating comparable baseline characteristics between cohorts. However, the hypophysitis group had a higher BMI than the control group. The median BMI was 26.92 kg/m² (IQR: 17.6, 39.1) in patients with hypophysitis, compared with 24.64 kg/m² (IQR: 16.7, 37.5) in the control group (P = 0.050) (**Table 2**). Chart review identified a small number of patients ultimately classified as having poorly differentiated carcinoma of unknown primary origin (n = 2 in the hypophysitis group and n = 6 in the control group), which did not affect the overall cancer-type distribution. The hypophysitis group exhibited a significantly lower mortality rate compared with the non-hypophysitis group (23.1% [6/26] vs. 48.2% [27/56], P = 0.031) (**Table 2**).

**Table 2:** Baseline characteristic.

Variable	Hypophysitis	Control	*P*
Patients	26	56	–
Gender			0.499
Male	10	26	
Female	16	30	
Race			0.505
White	25	51	
Asian	0	1	
Black	1	2	
Unavailable	0	2	
Ethnicity			0.836
Not Hispanic	26	54	
Hispanic	0	2	
Age			
Median (IQR), year	64 (46, 85)	67.5 (30, 83)	0.601
BMI			
Median (IQR), kg/m^2^	26.92 (17.6, 39.1)	24.64 (16.7, 37.5)	0.050
Cancer type			0.420
Lung Cancer	13	36	
Breast Cancer	2	4	
Melanoma	9	10	
Poorly Differentiated Carcinoma, Unknown Primary Cancer	2	6	
PD-1 inhibitors			0.203
pembrolizumab	17	44	
nivolumab	9	12	
Diseased			0.031
Yes	6	27	
No	20	29	

### Clinical and endocrine characteristics of hypophysitis

**Table 3** shows the clinical presentation was heterogeneous, most commonly characterized by fatigue (57.7%, 15/26), followed by nausea (30.8%, 8/26), hyponatremia (30.8%, 8/26), and fever (15.4%, 4/26).

**Table 3:** The clinical features of hypophysitis.

Parameter	*n*	(%)
Symptoms		
Hypotension	3	11.54
Fatigue	15	57.69
Lethargy	1	3.85
Muscle aches/cramps	3	11.54
Joint ache	1	3.85
Poor appetite	1	3.85
Nausea	8	30.77
Vomit	4	15.38
Diarrhea	2	7.69
Abdominal pain/discomfortable	2	7.69
Anorexia	1	3.85
Polydipsia	1	3.85
Mental status change	1	3.85
Fever	4	15.38
Chill	1	3.85
Headache	2	7.69
Dizziness	4	15.38
Median cycles to onset NO. (range)	9 (2-29)	
Pituitary hormone disturbance		
ACTH (7.2-63.0pg/ml)		
<5	19	73.08
7.2-63.0	5	19.23
>63.0	2	7.69
Cortisol(6-10AM): 6.0-18.4 ug/dL		
Below 0.2	5	19.23
0.2-6	17	65.38
6.0-18.4	2	7.69
>18.4	2	7.69
Concurrent with central Hypothyroidism	6	23.08
Concurrent with PD-1 associated Thyroiditis	3	11.54
Concurrent with central Hypogonadism	0	0.00
Concurrent with central diabetes Insipidus	1	3.85
Other disturbance		
Hyponatremia	8	30.77
MRI findings		
Normal	21	80.77
Enhancement of the pituitary gland or Pituitary stalk	2	7.69
Heterogeneous enhancement	1	3.85
Atrophy	1	3.85
Abnormal of posterior pituitary	0	0.00
Others: Interval enlargement.	1	3.85

The distribution of morning (6–10 AM) cortisol levels in the hypophysitis group indicated a high prevalence of adrenal insufficiency. The majority of patients (84.6%, 22/26) had cortisol concentrations below the reference range of 6.0-18.4 µg/dL, with 19.2% (5/26) demonstrating profoundly low levels below 0.2 µg/dL. Only 7.7% (2/26) were within the normal reference range, while an equal proportion (7.7%, 2/26) had concentrations above 18.4 µg/dL. Endocrine evaluation revealed ACTH deficiency (<5 pg/mL) in the majority of patients (73.1%, 19/26). Concurrent central hypothyroidism occurred in 23.1% (6/26), and 11.5% (3/26) had concurrent ICI-related thyroiditis. Central diabetes insipidus (3.9%, 1/26) was uncommon. No patient had clinically documented central hypogonadism; however, because gonadal-axis testing was not systematically performed in all patients, subclinical hypogonadism cannot be excluded.

Regarding imaging, most patients (80.8%, 21/26) had normal pituitary MRI findings on clinically obtained MRI studies performed as part of the diagnostic evaluation. A minority showed pituitary gland or stalk enhancement (7.7%, 2/26), heterogeneous enhancement (3.9%, 1/26), or pituitary atrophy (3.9%, 1/26). Exact MRI timing relative to biochemical diagnosis was not uniformly available due to the retrospective study design.

### Analysis of gene mutation profiles

To explore the association between tumor genomic alterations and susceptibility to PD-1 inhibitor-related hypophysitis, targeted next-generation sequencing data from tumor samples in both groups were analyzed. Mutation frequency analysis (**Table 4**) revealed a distinct tumor genomic profile in the hypophysitis group, which also exhibited a significantly higher tumor mutational burden (TMB; 6.02 vs. 5.19 mut/Mb, P = 0.002).

**Table 4:** Genetic mutation profiling analysis.

Genetic variables	Hypophysitis	Control	*P* ^*^
Total mutation count	692	1,284	–
Total locus number	11,492	24,752	–
Tumor mutational burden (TMB)	6.02%	5.19%	0.002
BABAM1			
Mutation/non-mutation	4/22	0/56	0.014
PAXIP1			
Mutation/non-mutation	0/26	11/45	0.037
KDM5C			
Mutation/non-mutation	4/22	0/56	0.014
CDH4			
Mutation/non-mutation	5/21	1/55	0.018
TAL1			
Mutation/non-mutation	5/21	1/55	0.018

*Continuous correction or Pearson chi-square test.

**Figure 2:**
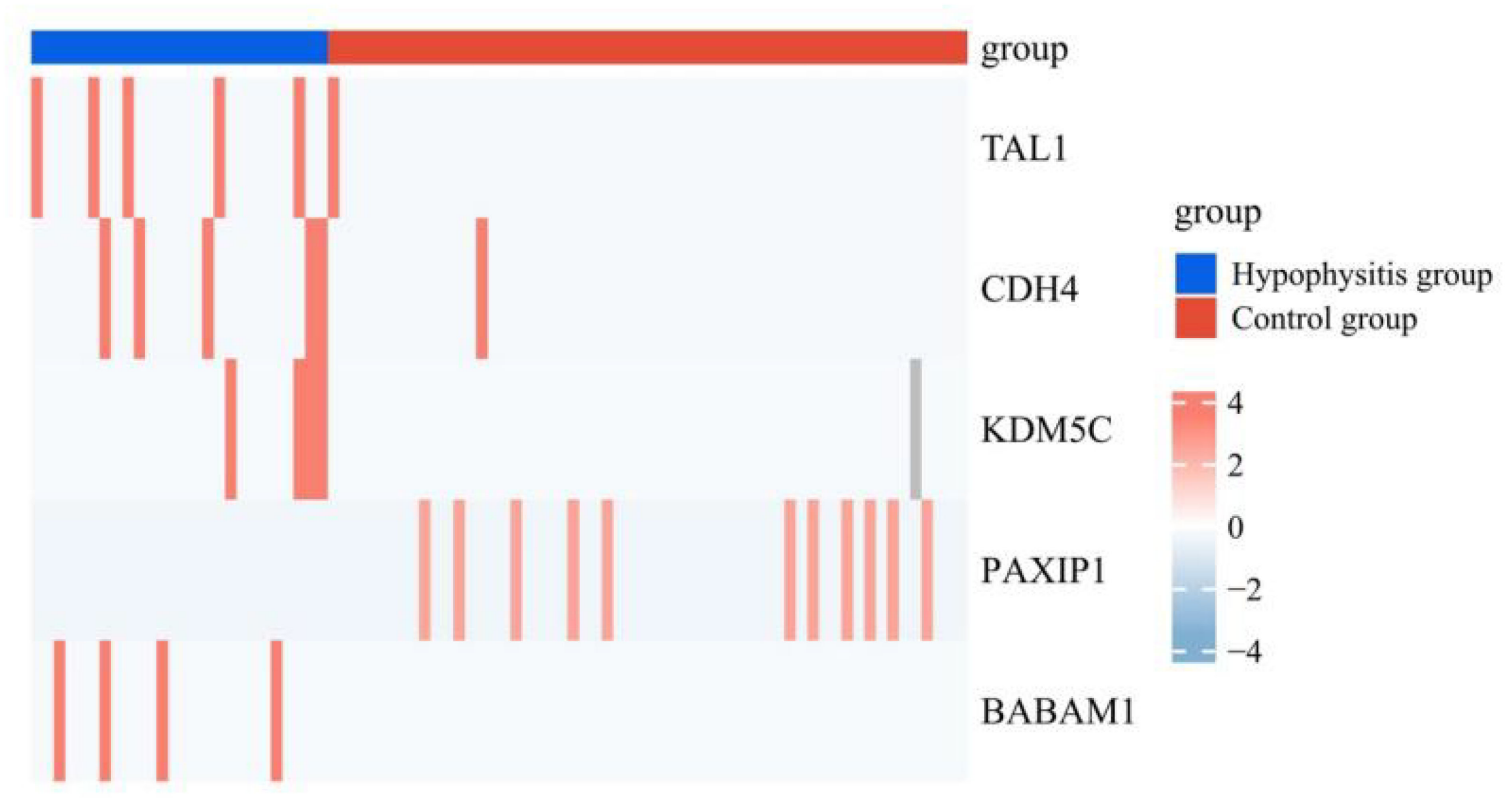
Heat map of genes mutation. The revised group annotation colors distinguish cohort membership from the mutation-prevalence gradient. The color gradient from blue to red corresponds to mutation prevalence, with darker red shades indicating higher mutation rates in the population.

Gene-specific comparisons further supported these findings. Mutations in BABAM1 (18.2% vs. 0%, P = 0.014), KDM5C (18.2% vs. 0%, P = 0.014), CDH4 (19.2% vs. 1.8%, P = 0.018), and TAL1 (19.2% vs. 1.8%, P = 0.018) were significantly enriched in the hypophysitis group, as illustrated in the heatmap (**Figure 2**) and confirmed in **Table 4**. Conversely, PAXIP1 mutations were significantly more prevalent in the non-hypophysitis group (19.6% vs. 0%, P = 0.037).

## Discussion

Despite durable anti-tumor responses mediated through T-cell activation, immune checkpoint inhibitors (ICIs) are associated with immune-related adverse events (irAEs), including potentially permanent endocrine toxicities (1, 2, 16). Among these, ICI-associated hypophysitis represents a clinically important complication, frequently leading to permanent hypopituitarism, most notably secondary adrenal insufficiency, which can be life-threatening if undetected or inadequately treated (7, 8). Early identification of patients at increased risk remains a critical unmet clinical need in immuno-oncology practice, and reliable predictive biomarkers for endocrine irAEs remain lacking.

Multiple mechanisms have been proposed to explain the development of irAEs, including tumor-self antigen cross-reactivity, host genetic susceptibility, pre-existing autoimmunity, and tumor immunogenicity (9, 17–21). Previous studies have predominantly focused on host-related factors. In contrast, the present study investigated whether tumor-intrinsic genomic characteristics are associated with PD-1 inhibitor-induced hypophysitis. We found that patients who developed hypophysitis exhibited higher TMB and a distinct tumor mutation profile, characterized by enrichment of BABAM1, KDM5C, CDH4, and TAL1 mutations, alongside a reduced frequency of PAXIP1 mutations.

Importantly, this study was designed as an exploratory association-based analysis of tumor genomic correlates of pituitary autoimmunity rather than a protein-functional study of individual variants. The central biological question is whether tumor mutation patterns reflect an immunogenic tumor state that increases the probability of endocrine autoimmunity after PD-1 blockade. Therefore, absence of variant-level protein prediction does not undermine the primary objective, although it limits mechanistic granularity. The identified mutations should not be interpreted as proven causal drivers of hypophysitis; rather, they may represent tumor genomic contexts associated with heightened immune activation under PD-1 inhibition.

Several enriched genes identified in the hypophysitis cohort, particularly BABAM1 and KDM5C, are involved in DNA damage response and chromatin regulation pathways. These pathways have been linked to enhanced tumor immunogenicity, elevated TMB, and increased responsiveness to immune checkpoint blockade (22–24). BABAM1 encodes a component of the BRCA1-A complex involved in homologous recombination-mediated DNA repair. Alteration of this pathway may promote genomic instability, micronuclei formation, cytosolic DNA accumulation, and innate immune sensing through pathways such as cGAS-STING. This mechanism could enhance type I interferon signaling, neoantigen presentation, and T-cell priming during PD-1 inhibition.

KDM5C encodes a histone H3K4 demethylase involved in chromatin remodeling and transcriptional regulation. KDM5C alterations have been associated with increased tumor immunogenicity, higher TMB, and stronger anti-tumor immune signatures (24). CDH4 and TAL1 are not classical DNA repair genes, but their enrichment suggests that multiple tumor pathways may converge toward an immunogenic phenotype. CDH4 encodes a cadherin family cell-adhesion molecule that may influence tumor-stromal interactions, endothelial permeability, and immune-cell trafficking within the tumor microenvironment. TAL1 is a transcription factor involved in hematopoietic and T-cell developmental programs. Although direct evidence linking CDH4 or TAL1 to pituitary-specific autoimmunity is limited, their enrichment supports a broader model in which diverse tumor genomic alterations may increase immune activation rather than directly injure pituitary cells.

Conversely, PAXIP1 mutations were less prevalent among patients with hypophysitis and were observed predominantly in the non-hypophysitis group. PAXIP1 participates in DNA damage signaling, chromatin regulation, and transcriptional control. Although this observation requires validation, it raises the possibility that PAXIP1-mutated tumors may be associated with a different immune contexture or reduced propensity for endocrine autoimmunity after PD-1 blockade. This finding should be regarded as a protective association signal rather than a validated protective biomarker.

Our data support a tumor immunogenicity-associated risk model for PD-1 inhibitor-induced hypophysitis. In this model, tumors with higher TMB and selected genomic alterations may generate increased neoantigen burden, greater release of damage-associated molecular patterns, and enhanced innate and adaptive immune activation. PD-1 blockade may then amplify anti-tumor immunity while increasing the risk of off-target endocrine autoimmunity. This mechanism could occur through molecular mimicry, shared or cross-reactive tumor-pituitary antigens, bystander immune activation, or loss of peripheral immune tolerance. Thus, the relevant biological link is not necessarily direct expression or functional disruption of these genes within the pituitary, but rather tumor-driven systemic immune activation that increases pituitary autoimmune vulnerability.

Clinically, these findings suggest that tumor genomic information collected for oncologic decision-making may also have value for endocrine toxicity risk stratification. Patients with higher TMB or tumors harboring immunogenic mutation profiles may warrant closer endocrine monitoring during PD-1 inhibitor therapy, including attention to fatigue, nausea, hyponatremia, hypotension, and early morning cortisol/ACTH abnormalities. However, these observations remain exploratory and should not yet be used to guide treatment selection or immunotherapy discontinuation without prospective validation.

Consistent with earlier reports, we observed lower mortality among patients who developed hypophysitis, supporting the hypothesis that endocrine irAEs may correlate with robust anti-tumor immunity and improved cancer outcomes (25–27). Furthermore, the predominance of normal pituitary MRI findings despite biochemical evidence of hypopituitarism reinforces that PD-1 inhibitor-associated hypophysitis frequently lacks classic radiologic features. Therefore, biochemical and clinical surveillance should remain central to diagnosis, and a normal MRI should not exclude PD-1 inhibitor-induced hypophysitis when ACTH deficiency is present (8, 28–31).

## Strengths and limitations

The primary strength of this study lies in its novel focus on tumor genomic correlates of endocrine irAEs, a largely understudied aspect of ICI safety. Nonetheless, the study has several limitations. First, the retrospective single-center design introduces the possibility of selection bias, incomplete data capture, and residual confounding. Although consecutive enrollment and matched cohort selection were used to minimize bias, unmeasured confounders, including genetic background, microbiome composition, concomitant medications, baseline autoimmunity, and other irAEs, cannot be completely excluded. Second, the relatively small sample size limits statistical power and increases the possibility of type II error. Third, paired germline sequencing was unavailable; therefore, the possibility that certain variants represent germline alterations rather than tumor-specific alterations cannot be excluded. Fourth, variant-level annotation, amino acid changes, variant allele frequencies, and population allele frequencies were not uniformly available from retrospective clinical reports, limiting protein-impact prediction and comparison with population databases. Finally, cosyntropin stimulation testing, gonadal-axis testing, MRI timing, and non-hypophysitis irAE ascertainment were not systematically collected. These limitations support the need for prospective multicenter studies with standardized endocrine phenotyping, paired germline sequencing, and integrated immunologic analyses.

## Conclusion

In summary, this exploratory study demonstrates an association between elevated TMB, distinct tumor genomic mutation profiles, and the development of PD-1 inhibitor-induced hypophysitis. Although these findings do not establish direct causality, they support the hypothesis that tumor-intrinsic immunogenicity contributes to pituitary autoimmunity and endocrine irAE susceptibility. Larger prospective multicenter studies incorporating standardized endocrine assessments, paired germline sequencing, variant-level annotation, and functional immune profiling are required to confirm the biological and clinical significance of these associations before clinical implementation.

## Data Availability

The original contributions presented in the study are included in the article/supplementary material. Further inquiries can be directed to the corresponding author.
